# Retraction Note: effectiveness of option B highly active antiretroviral therapy (HAART) prevention of mother-to-child transmission (PMTCT) in pregnant HIV women

**DOI:** 10.1186/1756-0500-7-868

**Published:** 2014-11-25

**Authors:** Erastus K Ngemu, Christopher Khayeka–Wandabwa, Eliningaya J Kweka, Joseph K Choge, Edward Anino, Elijah Oyoo-Okoth

**Affiliations:** School of Science, Department of Biochemistry, University of Eldoret, P.O Box 1125, Eldoret, Kenya; Institute of Tropical Medicine and Infectious Diseases (ITROMID), Jomo Kenyatta University of Agriculture and Technology (JKUAT), Nairobi, Kenya; Division of Livestock and Human Diseases Vector Control, Mosquito Section, Ngaramtoni, Tropical Pesticides Research Institute, Off Nairobi road, P.O. Box 3024, Arusha, Tanzania; School of Health Sciences, University of Eastern Africa, Baraton, P.O Box 2500–30100, Eldoret, Kenya; School Natural Resources and Environmental Studies, Karatina University, P.O. Box 1957–10101, Karatina, Kenya

## Retraction

This article [1] has been retracted by the editors of *BMC Research Notes* because, contrary to the statement in the manuscript, Moi University Institutional Research and Ethics Committee (IREC) has confirmed that ethics approval was not obtained to conduct this study. In addition, the authors did not have permission to use data from the DREAM program.

## References

[1] Ngemu EK, Khayeka–Wandabwa C, Kweka EJ, Choge JK, Anino E, Oyoo-Okoth E (2014). Effectiveness of option B highly active antiretroviral therapy (HAART) prevention of mother-to-child transmission (PMTCT) in pregnant HIV women. BMC Res Notes.

